# Do altruistic tendencies lead to the persistence of voluntary behavior? A moderated mediation analysis

**DOI:** 10.3389/fpsyg.2025.1553888

**Published:** 2025-04-02

**Authors:** Shujie Yuan, Zengzhen Zhao

**Affiliations:** ^1^Department of Psychology, Huangshan University, Huangshan, China; ^2^Student Affairs Department, Beijing Normal University, Beijing, China

**Keywords:** altruistic tendency, persistence of voluntary behavior, affective organizational commitment, psychological collectivism, relationship

## Abstract

**Background:**

In the postpandemic period, more attention has been given to the sustainability of volunteering in China. Do altruistic tendencies lead to the persistence of voluntary behavior in collectivistic culture? This study examined the relationship between altruistic tendencies and the persistence of voluntary behavior as well as the mediating effect of affective organizational commitment and the moderating effect of psychological collectivism.

**Methods:**

A two-wave study was conducted among 423 young volunteers in the Yangtze River Delta from February through April 2024. Model 4 and Model 7 from the SPSS macro PROCESS were used to test the model.

**Results:**

The sample data collected supported all the hypotheses. Specifically, the findings revealed that altruistic tendencies positively predicted the persistence of voluntary behavior among young volunteers. Affective organizational commitment partially mediated the relationship between altruistic tendencies and the persistence of voluntary behavior. Psychological collectivism positively moderated the relationship between altruistic tendency and affective organizational commitment and played a positive moderating role in the first half of the mediating path.

**Discussion:**

This study tested a moderated mediation framework in collectivistic culture. It revealed the mechanism underlying the influence of altruistic tendencies on the persistence of voluntary behavior, thus identifying important implications concerning the effective guidance of voluntary behavior and volunteer management.

## Introduction

Volunteer service is an important symbol of social engagement and progress in modern society, and an increasing number of people, especially young people, are engaging in volunteer work. The report of the 20th National Congress of the Communist Party of China proposed improvements to the volunteer service system and work system. Voluntary behavior refers acts by individuals or organizations that voluntarily contribute resources, abilities, and time without receiving compensation in the fields of social welfare, disaster prevention and reduction, and emergency rescue (Luo and Yu, 2016). The persistence of such voluntary behavior is crucial for promoting the high-quality development of volunteer services in China. In this context, studies have reported findings such as weak persistence of voluntary behavior and high turnover rates among volunteers (Zheng et al., 2020). The reasons for the interruption of voluntary behavior include individual factors such as demographic characteristics, personal traits, and psychological perceptions, as well as organizational factors such as institutional norms and transparency. Some scholars have claimed that the stronger the role identity of volunteers is, the more likely they are to participate in volunteer service for a long period of time (Albert et al., 2000; Emma et al., 2024). Luo examined a volunteer teaching project and reported that the stronger young volunteers’ role identity as “volunteers” and their sense of identification with the teaching project were, the more willing they were to continue participating in the project (Luo and Yu, 2016). When a person finds that volunteer service helps improve their abilities and promote career development, he or she continues to volunteer; similarly, if the volunteer work in which he or she engages involves rewards and praise, the likelihood of his or her continued engagement in volunteer service increases greatly. The reasons that young volunteers discontinue such service activities may be a failure of regulation or uneven social exchange (Zhang and Wu, 2015). The more satisfied volunteers are, the better the service they provide and the longer they continue to engage in this activity (Clary et al., 1998), whereas under other circumstances, they may withdraw from the activity (Willems et al., 2012). Penner’s comprehensive model of voluntary behavior suggests that trait variables are stable influencing factors that “determine whether a person becomes a volunteer,” whereas personal trait factors affect not only people’s initial voluntary behavior but also their continuous voluntary behavior, albeit to a lesser extent (Penner, 2002). A prosocial personality is closely related to the sustainability of volunteer services (Zheng et al., 2020). As a behavioral tendency that encourages individuals to sacrifice their own interests for the benefit of others (Kerr et al., 2004), an altruistic tendency has become the most critical demand in the context of volunteer service (Frisch and Gerrard, 1981). According to the theory of planned behavior, a person may exhibit strong behavioral intentions, but those intentions may not result in actual behavior (Harris and Hagger, 2007). Affective organizational commitment, which is a type of organizational commitment, pertains to an individual’s degree of involvement in the organization and participation in organizational social interactions, which often have significant and lasting effects on a wide range of individuals’ performance and behaviors (Gao-Urhahn et al., 2016). Psychological collectivism indicates that individuals are more willing to accept collective goals and social norms and prefer to work in collectives and that individuals who exhibit higher levels of psychological collectivism attach more importance to their social roles and pay more attention to others (Ng and Lucianetti, 2015). This study aims to explore the influence mechanism and boundary conditions pertaining to the effect of altruistic tendencies on the persistence of voluntary behavior in light of the roles of affective organizational commitment and psychological collectivism in this context. Its goal is to enrich the theory of volunteer service with Chinese characteristics and provide references that can constitute effective guidance for volunteer service management.

## Literature review and hypotheses

As a prerequisite for altruistic behavior, an altruistic tendency reflects a person’s initiative and willingness to help others. Individuals who exhibit strong altruistic tendencies often also exhibit a strong sense of social responsibility and a desire to help others, thus giving rise to the possibility of highly altruistic behavior (Elster, 2006). In addition, a prosocial value orientation has a significant predictive effect on the voluntary behavior of young employees (Wang et al., 2016). The personality trait of agreeableness affects volunteers’ service behavior (Carlo et al., 2015). Whereas altruistic motivation can effectively predict voluntary behavior, it does not significantly predict the duration of volunteer participation (Mowen and Sujan, 2005). Although inconsistencies may emerge between attitudinal tendencies and behaviors, actual voluntary behavior may not reflect altruistic tendencies (Schwartz and Tessler, 1972). Young volunteers in China generally exhibit positive prosocial tendencies, and individuals who exhibit strong altruistic tendencies often have higher levels of psychological capital and may participate in volunteer service more frequently, thus becoming long-term volunteers (Caprara et al., 2012). Owing to an individual’s altruistic tendencies, that individual’s initial voluntary behavior gradually gains the support and encouragement of others in the vicinity, thus leading to a stronger identification with the volunteer role. Accordingly, such an individual is more likely to continue to engage in volunteer service. Therefore, we empirically test the following hypothesis:

*H1*: Altruistic tendencies have a significant positive effect on the persistence of voluntary behavior.

Affective organizational commitment reflects an individual’s sense of unity with organizational goals and values as well as a psychological attachment to the organization, which is the most spontaneous and positive form of organizational commitment (Meyer and Allen, 1997). Individuals who exhibit strong altruistic tendencies are more willing to devote time, energy, and resources to others and are more likely to experience a strong sense of belonging to and thus identify with the organization, as they believe that their efforts can benefit others. In addition, individuals who exhibit altruistic tendencies are more likely to receive recognition and praise from their leaders and colleagues, thus increasing their job satisfaction and sense of work value. Owing to their deep emotional attachment to the organization, individuals who experience a strong sense of organizational commitment also identify with organizational goals and values more strongly and are more willing to invest additional effort in serving the organization (Yuan et al., 2024). Therefore, individuals who exhibit such commitment are most likely to support and voluntarily act to benefit the organization (Montani et al., 2017; Zatzick et al., 2015). When a person makes a strong emotional commitment to an organization, that person is more likely to remain in the organization for a long period of time. Volunteers’ sense of identity and emotional attachment to volunteer organizations can increase their acceptance of the values and goals of those organizations and satisfy their need for belonging, which in turn can increase their willingness to invest continuous effort as a member of the volunteer service (McCunn and Gifford, 2014). Therefore, we empirically test the following hypothesis:

*H2*: Affective organizational commitment plays a significant mediating role in the relationship between altruistic tendencies and the persistence of voluntary behavior.

To account for the influence of cultural values on individuals (Farh et al., 2007), this study introduces the variable of psychological collectivism to explain the persistence of voluntary behavior. Psychological collectivism, which is derived from collectivism at the social and cultural levels, reflects an individual’s cognitive judgments regarding and behavioral intentions toward collectives and member relationships. Individuals who exhibit high levels of psychological collectivism tend to prefer collective life, abide by collective norms, and experience a stronger sense of dependence (Wagner, 1995); thus, they tend to define interpersonal relationships in terms of “harmony” (Jackson et al., 2006). Individuals who exhibit higher levels of interdependent self-construal value their social roles more highly, pay more attention to others, and tend to establish friendly and harmonious interpersonal relationships with others. In the context of Chinese collectivist culture, individuals who exhibit high levels of psychological collectivism pursue positive interactions with others more avidly, care more about social evaluations, and seek to behave in ways that meet social expectations. A strong sense of psychological collectivism on the part of individuals indicates a higher degree of fit between the person in question and organizational values, and the person’s behavior is more likely to be influenced by their own responsibilities and organizational norms (Ng and Lucianetti, 2015). Owing to their dependence on and value identification with the organization, such individuals are more willing to contribute actively to the achievement of organizational goals and exhibit a higher level of affective organizational commitment. Therefore, we empirically test the following hypothesis:

*H3*: Psychological collectivism positively moderates the relationship between altruistic tendencies and affective organizational commitment.

On this basis, this study proposes a moderated mediation model, according to which the mediating role of affective organizational commitment is moderated by psychological collectivism. Affective organizational commitment influenced by altruistic tendencies can significantly affect the persistence of voluntary behavior, and psychological collectivism may play a positive moderating role in the process through which altruistic tendencies influence the persistence of voluntary behavior through affective organizational commitment.

**Figure 1:**
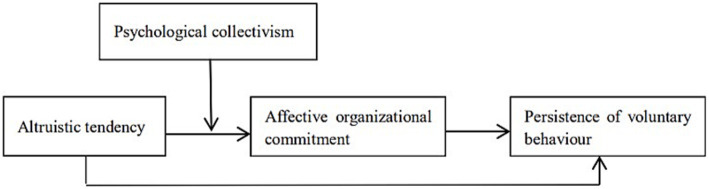
The proposed research model. Source: own elaboration.

The theoretical model for this research is illustrated in Figure 1.

## Method

### Participants and data collection

The current study was ethically approved by Beijing Normal University, China. After permission was obtained, the participants were briefed about the study and their ethical rights. They were instructed to approach the researchers in case of any ambiguity while completing the questionnaires. Then, the participants were presented with the questionnaire and were provided the instructions for completing the questionnaire. The participants completed the questionnaire in 10–15 min. At the end, the participants were thanked for their participation. This study adopted a two-wave design and was conducted in the Yangtze River Delta region of China from February through April 2024. The participants were informed of the study via a WeChat group and then received an invitation that described the aims, risks, benefits and process of the study; emphasized confidentiality; specified the participation requirements; and provided a link to the survey. A total of 550 participants consented to participate in the first survey. At Time 1, data on altruistic tendencies, affective organizational commitment, and psychological collectivism were collected. We received 462 valid responses, resulting in an 84% response rate. Two months later, at Time 2, a persistence of voluntary behavior scale was administered, and the second survey was answered by 423 out of the initial 462 respondents, resulting in a 91.56% response rate. Among the respondents, 28.6% were male, whereas 71.4% were female. A total of 60.8% of the respondents volunteered 1–10 times per year; 20.8%, 11–20 times per year; 10.2%, 21–30 times per year; and 8.3%, more than 30 times per year.

### Measures

Mature scales that exhibited high reliability and validity and that were developed by both domestic and foreign scholars were adopted. Except the measure of psychological collectivism, which was scored on a 7-point Likert scale, the other measures were scored on a 5-point Likert scale ranging from 1 = “completely disagree” to 5 = “completely agree.” Altruistic tendency was measured via the Altruistic Tendency Scale, which was developed by Clary et al. (1998). This scale contains 5 items. A typical item is “I always sympathize with people who need help.” Confirmatory factor analysis was performed on the scale, and the indicators fit well, with *χ*2/df = 3.32, RMSEA = 0.11, SRMR = 0.05, CFI = 0.94, and TFI = 0.87; the Cronbach’s alpha was 0.80, indicating good reliability and validity. The persistence of voluntary behavior was measured via the Voluntary Behavior Persistence Scale, which was developed by Deng (2018). This scale contains 5 items. A typical item is “I plan to participate in volunteer service for a long time.” Confirmatory factor analysis was performed on the scale, and the indicators fit well, with *χ*2/df = 2.94, RMSEA = 0.09, SRMR = 0.05, CFI = 0.92, TFI = 0.86, and Cronbach’s alpha = 0.81, indicating good reliability and validity. Affective organizational commitment was measured via the Affective Organizational Commitment Scale (Gao-Urhahn et al., 2016). The scale contains 5 items. A typical item is “The volunteer organization to which I belong is a great organization.” Confirmatory factor analysis was performed on the scale, and the indicators fit well, with *χ*2/df = 2.43, RMSEA = 0.08, SRMR = 0.02, CFI = 0.98, and TFI = 0.96, and the Cronbach’s alpha was 0.91, indicating good reliability and validity. Psychological collectivism was measured via the Psychological Collectivism Scale (Ng and Lucianetti, 2015). This scale contains 8 items. A typical item is “I work better alone than on a team.” Confirmatory factor analysis was performed on the scale, and the indicators fit well, with *χ*2/df = 3.81, RMSEA = 0.12, SRMR = 0.07, CFI = 0.90, and TFI = 0.83, and Cronbach’s alpha = 0.80, indicating good reliability and validity.

### Analytical approach

SPSS 22.0 software was used for data processing. The main methods employed included reliability analysis, descriptive statistics, and correlation analysis. With respect to the structural equation model, the PROCESS plugin was used to test the mediation and moderated mediation models in further detail with reference to the judgment methods suggested by Baron and Kenny (1987) and Wen and Ye (2014). Given the possible problem of common method bias in self-evaluation reports, Harman’s single-factor test method was used to examine the factors of all the items. This test revealed that 5 factors had eigenvalues greater than 1 and that the maximum factor explained 26.581% of the variance, i.e., less than 40% (critical value). Accordingly, no significant common method bias effect was observed in this study.

### Analysis of the results

The calculations enabled us to obtain participants’ scores corresponding to altruistic tendency (4.040 ± 0.334), the persistence of voluntary behavior (3.584 ± 0.323), affective organizational commitment (4.159 ± 0.400), and psychological collectivism (5.075 ± 0.717). A correlation analysis was performed to investigate the four variables. The results revealed that the correlation coefficients between altruistic tendencies and the persistence of voluntary behavior, affective organizational commitment, and psychological collectivism were 0.194, 0.348 and 0.049, respectively. The correlation coefficients between the persistence of voluntary behavior and both affective organizational commitment and psychological collectivism were 0.161 and 0.011, respectively. The correlation coefficient between affective organizational commitment and psychological collectivism was 0.128, as shown in Table 1. These results indicate that the prerequisites for Hypotheses 1 and 2 were met.

**Table 1:** Descriptive statistics and correlations (*N* = 423).

Variable	Mean	SD	1	2	3	4	5	6
1.Gender	1.710	0.452	1					
2.Volunteer frequency	1.390	0.729	−0.060	1				
3.Altruistic tendency	4.040	0.334	0.001	0.024	1			
4.Persistence of voluntary behavior	3.584	0.323	−0.038	0.089	0.194^***^	1		
5.Affective organizational commitment	4.159	0.400	−0.070	0.113^*^	0.348^***^	0.161^**^	1	
6.Psychological collectivism	5.075	0.717	0.024	0.071	0.049	0.011	0.128^**^	1

SD, standard deviation. Gender was coded as 1 = male and 2 = female. Volunteer frequency was coded as 1 = 1–10 times per year, 2 = 11–20 times per year, 3 = 21–30 times per year, and 4 = more than 30 times per year. *** *p* < 0.001, ** *p* < 0.01 (two-tailed).

Using the SPSS macro (Model 4) developed by Hayes, the mediating effect of affective organizational commitment on the relationship between altruistic tendencies and the persistence of voluntary behavior was tested, as shown in Table 2. The results of the regression analysis indicated that altruistic tendencies significantly and positively predicted the persistence of voluntary behavior (*β* = 0.194, *p* < 0.001), thus confirming H1. Additionally, altruistic tendencies significantly and positively predicted affective organizational commitment (*β* = 0.348, *p* < 0.001). When both altruistic tendencies and affective organizational commitment were included, the coefficient of altruistic tendencies on persistence of voluntary behavior decreased but remained significant (*β* = 0.171, *p* < 0.01), thus suggesting that affective organizational commitment partially mediated the relationship between altruistic tendencies and the persistence of voluntary behavior. Furthermore, when the bootstrap method was used with 5,000 resamples, the indirect effect value of affective organizational commitment was 0.041, and the bootstrap 95% confidence interval [0.002, 0.082] did not include zero, thus indicating that the mediating effect reached the level of significance. The calculation revealed that the proportion of the mediating effect to the total effect was 19.34%, as shown in Table 3, thus confirming H2.

**Table 2:** Regression analysis of the model variables.

**Regression model**		Comprehensive indicators		Regression coefficient	
Dependent variable	Independent variable	R^2^	F (df)	B (β)	*t*
Persistence of voluntary behavior	Altruistic tendency	0.037	16.399***(421)	0.212 (0.194)	4.050***
Affective organizational commitment	Altruistic tendency	0.121	58.144***(421)	0.404 (0.348)	7.625***
Persistence of voluntary behavior	Altruistic tendency	0.048	10.473***(420)	0.171 (0.156)	3.079**
	Affective organizational commitment			0.101 (0.107)	2.101**

**Table 3:** Test of the mediating effect via the bootstrap approach.

Effect type	*b*	Boot SE	Boot 95% CI		Relative effect
			Lower	Upper	
Total effect	0.212	0.052	0.109	0.315	
Direct effect	0.171	0.056	0.062	0.281	80.66%
Indirect effect	0.041	0.020	0.002	0.082	19.34%

The moderated mediating effect was examined using SPSS software (Model 7). The results first confirmed that the interaction term between altruistic tendencies and psychological collectivism exhibited a significant positive relationship with affective organizational commitment (*β* = 0.285, *p* < 0.001), with a 95% confidence interval of [0.125 ~ 0.445], thus indicating that the prerequisite for the subsequent analysis was satisfied.

**Figure 2:**
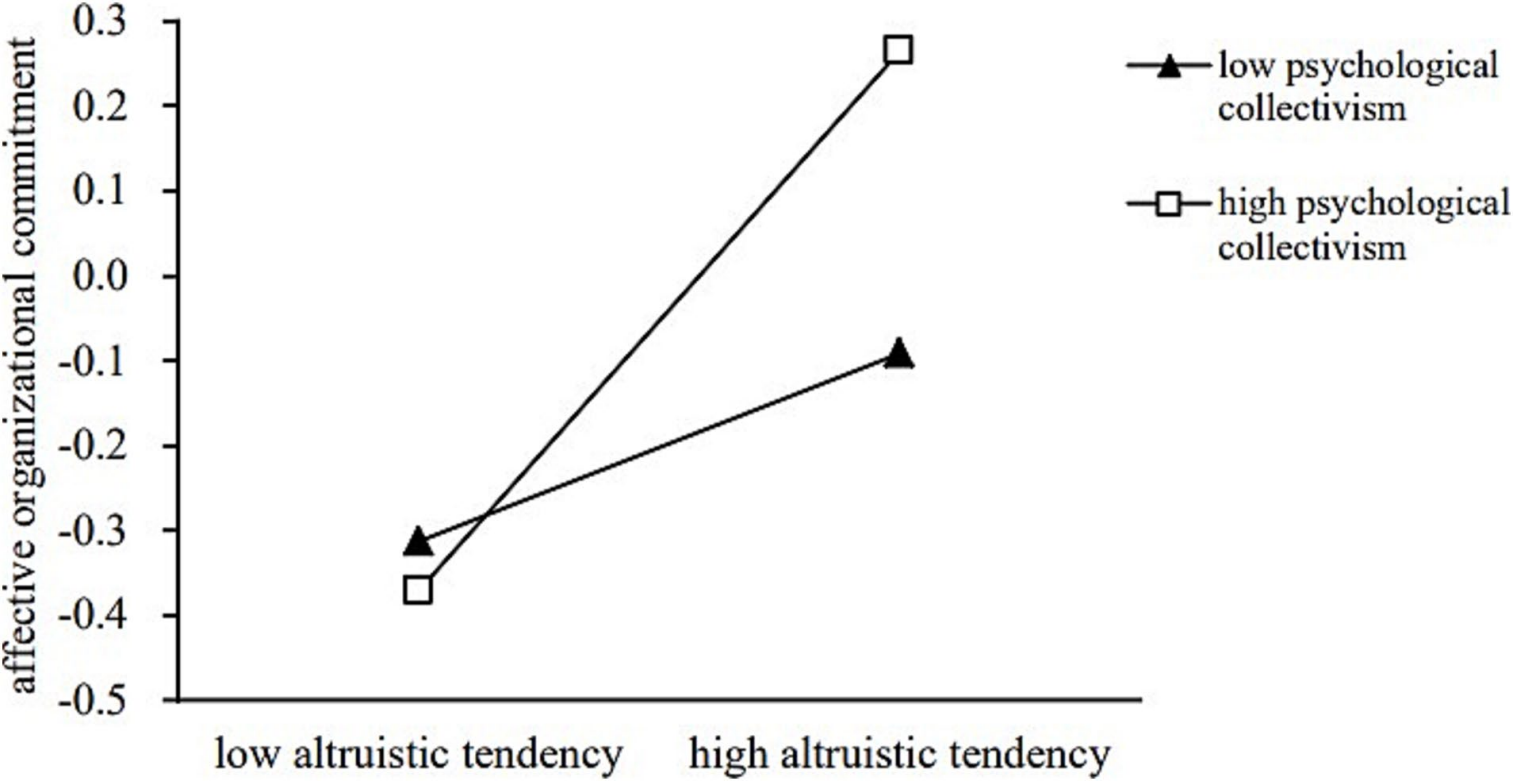
Moderating effect of psychological collectivism.

**Figure 3:**
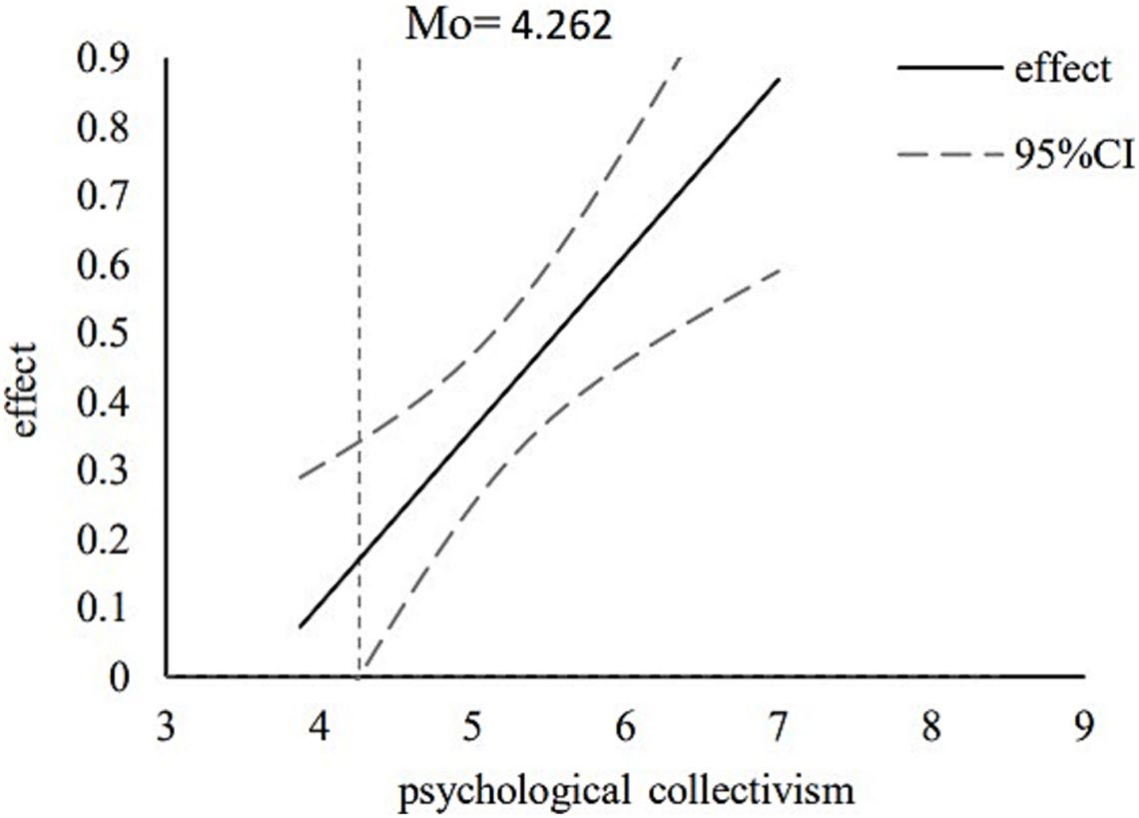
Trajectory of change in a simple slope.

In addition, the conditional indirect effects of the moderating variable at different values were analyzed via the pick-a-point approach. Specifically, a standard deviation was added and subtracted from the mean of the moderating variable to generate high and low values, as shown in Figure 2. The results indicated that when young volunteers exhibited a lower level of psychological collectivism, the impact of altruistic tendencies on the persistence of voluntary behavior was relatively small, with *b* = 0.019 and a bootstrap 95% confidence interval of-0.013 to 0.049. However, when young volunteers exhibited a higher level of psychological collectivism, the impact of altruistic tendencies on the persistence of voluntary behavior was greater, with *b* = 0.055 and a bootstrap 95% confidence interval of 0.002 to 0.110. Since one of these confidence intervals included zero, whereas the other did not, these results suggest that psychological collectivism moderated the first half of this mediation path, thus confirming H3. Considering that the pick-a-point method cannot properly account for the continuous process of moderating effects (Hayes, 2015), this study used the Johnson–Neyman method to calculate the 95% confidence band, and the value of the significance region and graphically shows the conditional indirect effect of the moderator variable under different values. When the value of psychological collectivism is greater than 4.262, the effect of altruistic tendencies through psychological collectivism is significant for the persistence of voluntary behavior, as shown in Figure 3. Furthermore, the INDEX value obtained from the PROCESS calculations was 0.029, with a bootstrap 95% confidence interval of 0.001 to 0.070, as shown in Table 4, thus indicating that the moderated mediating effect was significant. Accordingly, the results fully support H4.

**Table 4:** Analysis of the moderated mediating effect.

Dependent variable	Moderated indirect effect				Moderated mediation effect		
	Psychological collectivism	*b*	*SE*	BCa CI	INDEX	*SE*	BCa CI
Affective Organizational Commitment	Low value	0.019	0.013	[−0.013, 0.049]	0.029	0.017	[0.001, 0.070]
	Median value	0.037	0.019	[0.002, 0.075]			
	High value	0.055	0.027	[0.027, 0.110]			

## Discussion

This study revealed that altruistic tendencies have a significant positive predictive effect on the persistence of voluntary behavior. This research explores the path of “altruistic tendency → affective organizational commitment → persistence of voluntary behavior” and identifies psychological collectivism as an important moderator of this path. The findings of this research help to open the “black box” of how altruistic tendencies influence the continuity of voluntary behavior, namely, revealing that these tendencies can promote the persistence of voluntary behavior indirectly through affective organizational commitment. Even after the interference of affective organizational commitment and psychological collectivism is excluded, altruistic tendencies still have a significant positive predictive effect on the persistence of voluntary behavior. A possible explanation for this finding is that volunteers do not distinguish between these two types of motivation; rather, they coexist (Sun, 2012). Both motivation function theory and hierarchy of needs theory explain specific behaviors in terms of the satisfaction of psychological needs. As an individual’s level of involvement increases, volunteers’ altruistic motivation shifts from “external to internal” (Jing, 2010). As a traditional motivators of voluntary behavior, altruistic tendencies can enhance young volunteers’ emotional identification and compliance with organizational norms and goals; such tendencies can also fulfil psychological needs pertaining to value realization, thereby promoting more sustainable voluntary behavior. Therefore, it is necessary to strengthen the cultivation of empathy among young volunteers, actively establish a social support system of mutual assistance, provide education on socialist core values, and strengthen the cultivation of the quality of “kindness,” thereby making sustained voluntary behavior a social norm and moral belief that is recognized in mainstream values.

Affective organizational commitment plays a partial mediating role in the relationship between altruistic tendencies and the persistence of voluntary behavior, which verifies the bridging role of individuals’ attachment to and identification with the organization in the relationship between altruistic tendencies and the persistence of voluntary behavior. That is, altruistic tendencies can influence the persistence of voluntary behavior indirectly through affective organizational commitment. Self-determination theory posits that individuals continuously perform important tasks when they are spiritually energized to pursue valuable goals and are in an environment that facilitates autonomous action (Deci and Ryan, 2008). This study supports this claim in the field of volunteer service, thereby enriching and expanding the findings of research on this topic. An altruistic tendency originates from an attitude that is formed through interactions between the environment and the individual; such an attitude is oriented toward a mode of action that focuses on helping others solve problems. This positive tendency to help others promotes interpersonal harmony, thus making it easier for individuals in organizations to receive affirmation and recognition, fostering identification with organizational goals and compliance with organizational norms. This tendency thus represents an important prerequisite for engaging in socially desirable volunteer services for a long period of time. Therefore, providing active guidance to young volunteers is essential to help them recognize the value of voluntary behavior, understand challenges through organizational goals and feedback‌, align with norms and engage fully in activities‌. This approach can enable young volunteers to experience responsibility and mission-driven commitment‌ (Zhang et al., 2022), strengthen emotional identification with volunteer roles‌, establish diversified participation channels for sustainable engagement, improve behavioral outcome feedback mechanisms‌, and lay a foundation for high-quality volunteer service development‌.

This study revealed that psychological collectivism positively moderates the path of “altruistic tendency → affective organizational commitment → persistence of voluntary behavior.” Specifically, the higher the level of psychological collectivism is, the greater the influence of altruistic tendencies on the persistence of voluntary behavior via affective organizational commitment. In terms of Geert Hofstede’s cultural dimensions, China is a high-collectivism country. Individuals who exhibit a strong collectivistic tendency tend to emphasize their membership in an internal group and their loyalty to that group, to value positive interactions among individuals within the group, to establish a harmonious atmosphere, and to make positive contributions to the group (Zhong et al., 2016). Young volunteers are in a critical stage of self-concept development, where they must constantly construct their personal self, relational self, and collective self. Individuals who exhibit low levels of psychological collectivism tend to focus more on themselves, to consider others’ situations to a lesser degree, and to be less willing to participate actively in social interactions. This finding also illustrates the contextual role of the collective self and collectivistic tendencies in shaping prosocial behavior in China. Therefore, to strengthen young volunteers’ sense of collectivism, guide them to develop themselves within the collective, encourage them to engage in healthy interactions in a harmonious collective atmosphere, foster a spirit of collectivism, and enable them to experience the care and warmth of their fellow members through collective activities, various forms of “heart-warming projects” can be launched to increase mutual respect and love within the group, thus establishing an atmosphere that can increase sustainable volunteer behavior.

### Limitations and future research directions

This study explores the mechanism underlying the relationship between altruistic tendencies and the persistence of voluntary behavior in young volunteers, with affective organizational commitment as a mediator and psychological collectivism as a moderator. Although the data were collected at two stages, it would be better to measure altruistic tendencies and affective organizational commitment at two different times rather than at the same time. In addition, this study identified only psychological collectivism as an individual moderating variable. And the study focuses only on a group within a population having a collective upbringing and not on a population with an individualistic upbringing such as western societies. In the context of specific work practices, organizational factors, such as institutional norms and transparency (Clary and Snyder, 1999; Carlo et al., 2015), may also be relevant factors, as may volunteer trust and volunteer flaws (Rodell and Lynch, 2015). Voluntary behavior reflects the progress of social civilization, and the national “14th Five-Year Plan” identified “extensively carrying out volunteer service and care initiatives” as an important part of the goal of “continuously improving citizens’ cultural and ethical standards.” Therefore, in the future, a multilayer dynamic formation mechanism regarding the persistence of voluntary behavior can be constructed to explore more influencing factors, thereby providing a beneficial reference for volunteer organizations to implement voluntary behavior management in a scientific manner and promote the sustainable development of volunteer services more effectively.

## Conclusion

We demonstrate the link between altruistic tendencies and the persistence of voluntary behavior. We find a partial mediating role of affective organizational commitment in the relationship between altruistic tendency and the persistence of voluntary behavior. In addition, we find a positive moderating role of psychological collectivism in the mediated relationship of “altruistic tendency → affective organizational commitment → persistence of voluntary behavior.” We hope that this paper can inspire a fresh direction in the field and that can serve as a reference for researchers interested in voluntary behavior in Chinese culture.

## Data Availability

The original contributions presented in the study are included in the article/supplementary material, further inquiries can be directed to the corresponding author.
